# Validation of the French Translation of the Movement Disorder Society Non‐Motor Symptoms Scale (MDS‐NMS) in Parkinson's Disease

**DOI:** 10.1002/mdc3.70323

**Published:** 2025-09-01

**Authors:** Clément Desjardins, Stéphan Grimaldi, Sheng Luo, Luowen Yu, Christopher G. Goetz, Glenn T. Stebbins, Pablo Martinez‐Martin, Monica M. Kurtis, Tiago A. Mestre, Alvaro Sanchez‐Ferro, Michelle H.S. Tosin, Roberta Balestrino, Chi‐Ying R. Lin, Carmen Gasca‐Salas, Tatiana Witjas, Olivier Colin, David Maltete, Luc Defebvre, Caroline Giordana, Mahmoud Charif, Claire Thiriez, Chloé Laurencin, Mélissa Tir, Gwendoline Dupont, Philippe Remy, Christine Tranchant, Sophie Drapier, Alexandra Samier, Isabelle Benatru, Sara Sambin, Jean‐Christophe Corvol, Fatma Khelifi, Margherita Fabbri, Olivier Rascol, the NS‐Part Cohort study group and the MDS COA Translation Steering Committee, Jean‐Christophe Corvol, Olivier Rascol, Margherita Fabbri, Wassilios Meissner, Stéphane Thobois, Luc Defebvre, Helene Esperou, Florence Tubach, Yann de Rycke, Stephanie Carvalho, Yajiththa Rajasegaram, Layidé Roufai, Dalila Ouazib, Sandra Zarrad, Fatma Khelifi, Nacim Miri, Florence Tubach, Yann de Rycke, Nathalie Bertille, Vanessa Rousseau, Samuel Tessier, Margherita Fabbri, David Tavel, Dounya Metdaoui, Avigaelle Abitbol, Mathias Antunes, Alexis Brice, Suzanne Lesage, Christelle Tessson, Fanny Casse, Mélanie Ferrien, Guillaume Cogan, Lisa Welment, Silvia di legge, Melissa Tir, Bertrand Degos, Thierry Moulin, Wassilios Meissner, Claire Thiriez, Ana Marques, Philippe Remy, Gwendoline Dupont, Elena Moro, Luc Defebvre, Jean‐Luc Houeto, Stéphane Thobois, Jean‐Philippe Azulay, Christian Geny, Solène Frismand, Anne‐Gaëlle Corbille, Caroline Giordana, Giovanni Castelnovo, Jean‐Christophe Corvol, Isabelle Benatru, Anne Doe de Maindreville, Sophie Drapier, David Maltete, Christine Tranchant, Olivier Rascol, Mickael Aubignat, Marie Mongin, Arnaud Lapostolle, Matthieu Bereau, Gautier Clement, Alexandra Foubert‐Samier, Brice Laurens, Sylvain Vergnet, Thomas Boraud, David Bendetowicz, Jade Sarrabere, Dominique Guehl, Pierre Burbaud, Edouard Courtin, Paul‐Alexandre Pfeiffer, Gilles Defer, Bérengère Debilly, Philippe Derost, Charlotte Beal, Hayet Salhi, Alice Dormeuil, Aimée Petit, Alban Gravier, Vincent Schneider, Lucie Garnier, Nicolas Carriere, Olivier Colin, Philippe Couratier, Chloé Laurencin, Stephane Prange, Paul Jaulent, Bruno Plus, Helene Gervais‐Bernard, Alexandre Eusebio, Frédérique Fluchere, Stephan Grimaldi, Valentin Mira, Tatiana Witjas, Mahmoud Charif, Ophelie Forster, Alix Durand, Pauline Prin, Lucie Hopes, Amory Jardel, Salome Puisieux, Guillemette Clement, Philippe Damier, Tiphaine Rouaud, Pascal Derkinderen, Arthur Lionnet, Adrien de Lataillade, Cosmin Alecu, Charlotte Heraud, Marie de Verdal, Graziella Mangone, Sara Sambin, Aymeric Lanore, Thomas Courtin, Louise‐Laure Mariani, Fouad Khoury, Poornima Menon, Florence Cormier‐Dequaire, Emmanuel Flamand‐Roze, David Grabli, Elodie Hainque, Marie Vidhaillet, Aurélie Meneret, Cécile Delorme, Cendrine Foucard, Florian Von Raison, Alexis Elbaz, Andreas Hartmann, Vincent Leclercq, Theodore Soulier, Daniel Torres, Giulia Coarelli, Giorgia Querin, Fabien Hauw, Margaux Dunoyer, Solène Ansquer, Frederique Leh, Marion Leclercq, Simon Lamy, Guillaume Carey;Guillaume Costentin, Clemence Hardy, Mathieu Anheim, Ouhaid Lagha Boukbiza, Thomas Wirth, Jimmy Voirin, Marie Des Neiges Santin, Thomas Bogdan, Margherita Fabbri, Fabienne Ory‐Magne, Christine Brefel Courbon, Clemence Leung, Hélène Catala, Gabrielle Sill, Raquel Pinheiro Barbosa, Lucy Famer, Lucie Braccagni, Hiba Sifaoui, Juliette Palisson, Kenza Benrahmoune Idrissi, Astrid Causel, Lydie Romeo, Constance Bissessur, Audace Cure‐Martin, Charline Compagne, Sandrine Dupouy, Sandrine Villars, Wei‐Ho Lai, Rachida Bari, Damien Chevanne, Elodie Durand, Isabelle Rieu, Stephane Bernard, Corinne Garsault, Nathalie Meunier, Alexia Cresson, Marine Sgard, Marie Dreano, Justine Montillot, Renaud Massart, Pascale Grebent, Pierre Pelissier, Valérie Santraine, Thomas Gaudin, Pierre Boutet, Cécile Thuilier, Coralie Chalot, Céline Prevost, Hélène Videaud, Justine Picut, Christian Tarrade, Catherine Caire, Hélène Merle, Elise Metereau, Mathilde Millot, Chloe Bernardi, Emilie Favre, Adelaide Jaulent, Laura Mundler, Blandine Dufresne, Eve Benchetrit, Valérie Driss, Alexia Arifi, Maura Rodrigues, Lili Le Monnier, Nathalie Dumont, Virginie Bablon, Régis Frenais, Caroline Herve, Christelle Guimber, Vanessa Ferrier, Elodie David, Christina Faroul, Leslie Fra, Elsa Foucaran, Fatima‐Ezzahra Ennaji, Jeremy Bonetto, Ryad Ladghem‐Chikouche, Le Mickael, Sophie Liot, Sonia Messar, Hamza Salah, Amelie Bernardo, Naoual Serari, Emilie Rabois, Carole David, Zoe Fournier, Margaux Bonnaire‐Verdier, Elise Chevaillier, Françoise Kestens, Rozenn Gourhan, Sandra Lopez‐Alfaro, Jean‐François Houvenaghel, Mélanie Alexandre, Christine Bourdonnais, Linda Vernon, Ahmed Boumediene, Hugo Rummel, Céline Julie, Nadine Longato, Célie Phillipps, Anne Claire Andries‐Ros, Stéphanie Bras, Estelle Harroch, Claudia Gillet, Yoan Herades, Eva Camgrand

**Affiliations:** ^1^ Department of Neurology Foundation Rothschild Hospital, Paris Cité Université Paris France; ^2^ Department of Neurology and Movement Disorders AP‐HM, CHU La Timone Marseille France; ^3^ Duke University Durham NC USA; ^4^ Department of Neurological Sciences Rush University Medical Center Chicago IL USA; ^5^ Centro de Investigacion Biomédica en Red sobre Enfermedades Neurodegenerativas (CIBERNED) Madrid Spain; ^6^ Hospital Ruber Internacional Madrid Spain; ^7^ Parkinson's Disease and Movement Disorders Clinic, Division of Neurology, Department of Medicine The Ottawa Hospital Research Institute Ottawa ON Canada; ^8^ University of Ottawa Brain and Mind Institute Ottawa ON Canada; ^9^ Movement Disorders Unit, Neurology Department Hospital Universitario 12 de Octubre Madrid Spain; ^10^ Vita‐Salute San Raffaele University Milan Italy; ^11^ Neuroimaging Research Unit, Division of Neuroscience IRCCS San Raffaele Scientific Institute Milan Italy; ^12^ Neurology and Neurorehabilitation Unit IRCCS San Raffaele Scientific Institute Milan Italy; ^13^ Baylor College of Medicine Houston TX USA; ^14^ HM CINAC (Centro Integral de Neurociencias Abarca Campal) Hospital Universitario HM Puerta del Sur, HM Hospitales Madrid Spain; ^15^ School of Medicine University CEU‐San Pablo Madrid Spain; ^16^ Department of Neurology CH Brive, Limoges Parkinson Expert Centre, French NS‐Park/F‐CRIN Network Brieve France; ^17^ Department of Neurology, NS‐PARK/FCRIN Network Rouen University Hospital and University of Rouen Rouen France; ^18^ INSERM U1239, Laboratory of Neuronal and Neuroendocrine Differentiation and Communication Mont‐Saint‐Aignan France; ^19^ Movement Disorders Department Lille University Lille France; ^20^ Department of Neurology Centre Hospitalier Universitaire de Nice, Université Côte d'Azur Nice France; ^21^ Department of Neurology CHRU Montpellier Montpellier France; ^22^ Department of Neurology and Parkinson Expert Centre Caen University‐Hospital Caen France; ^23^ Department of Neurology C, Parkinson Expert Center Pierre Wertheimer Neurological Hospital, Hospices Civils de Lyon Bron France; ^24^ Department of Neurology University Hospital of Amiens Amiens France; ^25^ Département de Neurologie Centre Hospitalier Universitaire François Mitterrand, Université de Bourgogne Dijon France; ^26^ Centre Expert Parkinson, Neurologie, CHU Henri Mondor, AP‐HP, Equipe 01‐NPI, IMRB INSERM et Ecole Normale Supérieure, Faculté de Santé Université Paris‐Est Créteil Créteil France; ^27^ Service de Neurologie, NS‐PARK/FCRIN Network Hôpitaux Universitaires de Strasbourg Strasbourg France; ^28^ Department of Neurology, CIC 1414 Rennes University Hospital and NS‐Park/FCRIN Network Rennes France; ^29^ CHU Bordeaux, Service de Neurologie des Maladies Neurodégénératives, and NS‐Park/FCRIN Network IMNc Bordeaux France; ^30^ Department of Neurology University Hospital of Poitiers Poitiers France; ^31^ Sorbonne Université, Paris Brain Institute—ICM, Inserm, CNRS, Assistance Publique Hôpitaux de Paris, CIC Neusociences, Pitié‐Salpêtrière Hospital, France Sorbonne Université Paris France; ^32^ NS‐Park Network Toulouse France; ^33^ Department of Clinical Pharmacology and Neurosciences, Toulouse Parkinson Expert Centre, Toulouse NeuroToul Center of Excellence in Neurodegeneration (COEN), French NS‐Park/F‐CRIN Network University of Toulouse 3, CHU of Toulouse, INSERM Toulouse France

**Keywords:** Parkinson's disease, non‐motor symptoms, rating scale, MDS‐NMS, translation

The MDS‐Non‐Motor Symptoms Scale (MDS‐NMS) is a comprehensive tool to assess NMS in Parkinson's disease (PD), which represent a major source of disability and impact on quality of life throughout the disease course.^1^, ^2^ While validated in several languages,^3^, ^4^ a French version was lacking, limiting its use in French‐speaking populations worldwide. We followed the official Movement Disorder Society‐Clinical Outcome Assessment protocol to translate, adapt, and validate the French version, including forward–backward translation, expert review, and cognitive pretesting. Methodological details of the analyses are provided in Supplementary Material S1, including translation protocol, primary/secondary analysis and cognitive pretesting details.

A total of 303 PD patients were recruited from 10 expert centers within the NS‐Park French clinical research network. Inclusion criteria required a confirmed diagnosis of PD and high proficiency in French. Participants had a mean age of 62.3 years and mean disease duration of 8.2 years; most were in Hoehn & Yahr stage 2. Full demographic data are available in Supplementary Table S1.

Cognitive pretesting in 10 patients demonstrated the clarity and acceptability of the translated scale, with no item requiring rewording or removal. The scale was acceptable, culturally appropriate, and needed no major adaptation, allowing its approval as an “official working document” per MDS standards. This step ensured that the translation preserved both linguistic fidelity and clinical relevance, and could be implemented uniformly across clinical centers.

Confirmatory factor analysis (CFA) confirmed the structure of the original English version, with CFI values ≥0.90 for most subscales (Table 1). Domains with too few items—such as Apathy, Orthostatic Hypotension, Sexual, and Urinary—did not yield model fit indices due to statistical limitations. Exploratory factor analysis (EFA) revealed high item‐factor loadings (≥0.40), with dominant factors explaining substantial variance in key domains (eg, Depression: 57.2%, Cognition: 53%). Full EFA results and variance data are presented in Supplementary Table S2, and scree plots are shown in Supplementary Figure S1.

**TABLE 1:** French Confirmatory Factor Analysis (CFA) results for the 14 subscales (13 domains of the MDS‐NMS plus the NMF subscale)

Item	CMIN	DF	RMSEA	CFI
A. Depression	5.10	5	0.01	1.00
B. Anxiety	4.22	2	0.06	1.00
C. Apathy	–	–	–	–
D. Psychosis	9.28	2	0.11	0.99
E. Impulse Control and Related Disorders	4.99	2	0.07	0.98
F. Cognition	28.40	9	0.08	0.99
G. Orthostatic Hypotension	–	–	–	–
H. Urinary	–	–	–	–
I. Sexual	–	–	–	–
J. Gastrointestinal	0.04	2	0.00	1.00
K. Sleep and Wakefulness	29.13	9	0.09	0.92
L. Pain	3.32	2	0.05	1.00
M. Other	17.13	5	0.09	0.96
NMF (Fluctuations)	11.02	20	0.00	1.00

CMIN, χ^2^; DF, degrees of freedom; RMSEA, root mean square error of approximation; CFI, comparative fit index; NMF, Non‐Motor Fluctuation.

The MDS‐NMS covers 13 symptom domains and non‐motor fluctuations, enabling a detailed and multidimensional assessment of neuropsychiatric, autonomic, sleep‐related, sensory, gastrointestinal, and cognitive symptoms. This breadth is essential for understanding the full complexity of PD beyond motor features. Our findings align closely with prior validations in Portuguese and Spanish,^3^, ^4^ confirming the cross‐cultural robustness of its structure.

The French MDS‐NMS is particularly relevant for longitudinal studies, clinical trials, and real‐world evaluations of PD. It enables better characterization and follow‐up of NMS, which are often underrecognized and undertreated despite their burden. Its use may support more personalized care strategies and facilitate cross‐national research collaborations. Some domains—such as gastrointestinal and sleep‐wakefulness—showed more complex factor structures, as observed in other language validations, suggesting potential avenues for refinement. Future efforts may focus on validating a French patient‐reported version, as has been done for the English MDS‐NMS‐Q.^5^

In conclusion, the French MDS‐NMS is a valid, culturally adapted scale aligned with international standards. It represents a key step toward harmonizing the assessment of NMS in PD across language barriers and optimizing care for French‐speaking patients worldwide.

## Author Roles

At the end of the manuscript, list the itemized contributions in number/letter format, as below. These should include but are not restricted to: (1) Research project: A. Conception, B. Organization, C. Execution; (2) Statistical Analysis: A. Design, B. Execution, C. Review and Critique; (3) Manuscript Preparation: A. Writing of the first draft, B. Review and Critique.

C.D.: 1C, 2C, 3A, 3B.

S.G.: 1C, 2C, 3A, 3B.

S.L.: 2A, 2B, 2C, 3B.

L.Y.: 2A, 2B, 2C.

C.G.G.: 1A, 1B, 2C, 3B.

G.T.S.: 1A, 1B, 2C, 3B.

P.M.M.: 1A, 1B, 2C, 3B.

M.M.K.: 1A, 1B, 2C, 3B.

T.A.M.: 1A, 1B, 2C, 3B.

A.S.F.: 1A, 1B, 2C, 3B.

M.H.S.T.: 1A, 1B, 2C, 3B.

R.B.: 1A, 1B, 2C, 3B.

C.L.: 1A, 1B, 2C, 3B.

C.G.S.: 1A, 1B, 2C, 3B.

T.W.: 1A, 1B, 1C, 2C, 3B.

O.C.: 1A, 1B, 1C, 2C, 3B.

D.M.: 1A, 1B, 1C, 2C, 3B.

L.D.: 1A, 1B, 1C, 2C, 3B.

C.G.: 1A, 1B, 1C, 2C, 3B.

M.C.: 1A, 1B, 1C, 2C, 3B.

C.T.: 1A, 1B, 1C, 2C, 3B.

C.L.: 1A, 1B, 1C, 2C, 3B.

M.T.: 1A, 1B, 1C, 2C, 3B.

G.D.: 1A, 1B, 1C, 2C, 3B.

P.R.: 1A, 1B, 1C, 2C, 3B.

C.T.: 1A, 1B, 1C, 2C, 3B.

S.D.: 1A, 1B, 1C, 2C, 3B.

A.S.: 1A, 1B, 1C, 2C, 3B.

I.B.: 1A, 1B, 1C, 2C, 3B.

S.S.: 1C, 2C, 3B.

J.C.C.: 1C, 2C, 3B.

F.K.: 1A, 1B, 2C, 3B.

M.F.: 1A, 1B, 1C, 2C, 3B.

O.R.: 1A, 1B, 1C, 2C, 3B.

## Disclosures

**Ethical Compliance Statement:** According to the French ethic and regulatory law (public health code) retrospective studies based on the exploitation of usual care data do not require to be submitted at an ethics committee but they have to be declared or covered by reference methodology of the French National Commission for Informatics and Liberties (CNIL). The authors confirm that the approval of an institutional review board was not required for this work. All patients received written information about the study, and could express their opposition rights through the cohort website in accordance to EU General Protection Data Regulation rules (https://parkinsonnetwork/la-cohorte-ns-park) (https://parkinson.network/). A collection and computer processing of personal and medical date was implemented to analyze the results of the research. Toulouse University Hospital signed a commitment of compliance to the reference methodology MR‐004 of the French National Commission for Informatics and Liberties (CNIL). After evaluation and validation by the data protection officer and according to the General Data Protection Regulation*, this study completing all the criteria, it is register in the register of data study of the Toulouse University Hospital (number's register: RnIPH 2023‐39) and cover by the MR‐004 (CNIL number: 2206723 v 0). We confirm that we have read the Journal's position on issues involved in ethical publication and affirm that this work is consistent with those guidelines.

**Funding Sources and Conflicts of Interest:** No specific funding was received for this work. The authors declare that there are no conflicts of interest relevant to this work.

**Financial Disclosures for the previous 12 months:** CD received Honoraria to speak from Téva Santé and Expression Santé, consultancies for Téva Santé. GTS received consulting and advisory board honoraria from Alzheimer's Association, Critical Path Institute, International Parkinson and Movement Disorder Society, Huntington Study Group, Lilly USA, Michael J. Fox Foundation for Parkinson's Research, Neurocrine Biosciences, Inc., Octave Bioscience, Pfizer, Inc., Vima Therapeutics, Inc., WCG Clinical, Inc. He has received grants from Critical Path Institute, CHDI Management, Inc., International Parkinson and Movement Disorder Society, Michael J. Fox Foundation for Parkinson's Research, Ottawa Hospital Research Institute, University of California at San Diego, University of California at San Francisco. TAM received consulting and advisory board membership honoraria with Abbvie, International Parkinson and Movement Disorder Society, AbbVie, CHDI Foundation/Management, Merz, WAVE pharmaceutical, PTC therapeutics and Roche. He has research grants with EU Joint Program—Neurodegenerative Disease Research, uOBMRI, Roche, Ontario Research Fund, CIHR, Michael J. Fox Foundation for Parkinson's Research, Parkinson Canada, Parkinson Research Consortium and Brain Canada. He receives salary with University of Ottawa Medical Associates. RB received a travel grant from Lusofarmaco. Alvaro Sanchez‐Ferro received grants or contracts from MDS (eDiary project), and Instituto de Salud Carlos III (reference number P122/01177); consulting fees from AbbVie, Boston Scientific, Esteve, Orion Pharma, Prim; and Roche and payment or honoraria for lectures, presentations, speakers bureaus, manuscript writing, or educational events from AbbVie, Bayer, Esteve, MDS Society, EAN, Novartis, Monitor, Organon, Roche, SEN, Stada, Teva, and Zambon. Salary: Leuko Labs and Hospital 12 de Octubre. Royalties from Patents. Stock: Leuko Labs. MHST received salary—Rush University; Professional Societies: working group supported by the International Parkinson and Movement Disorder Society. CG‐S received consulting with honoraria: Instituto de Salud Carlos III. Grants: Community of Madrid Grant for Research Assistant. Funding to attend a scientific meeting: Esteve. CT has received honoraria or travel grants from AbbVie and Adelia. JCC has served in advisory boards for Alzprotect, Bayer, Ferrer, iRegene, Servier, UCB, Roche; and received grants from AXA and the ICM Foundation outside of this work. OR has acted as a scientific advisor for drug companies developing antiparkinsonian medications (Abbott, Abbvie, Acorda, Adamas, BIAL, Biogen, Boehringer‐Ingelheim, Cynapsus, GSK, Impax, Merck, Osmotica, Oxford‐Biomedica, Lundbeck, Novartis, Prexton, Servier, Sunovion, TEVA, UCB, Zambon). Margherita Fabbri received Honoraria to speak from AbbVie, ORKYN, and BIAL, consultancies from BIAL and LVL Medical; Grant from France Parkinson, HORIZON 2022 French Ministry of Health and MSA Coalition.

## Supporting information


**Figure S1.** Scree plots for each domain of the MDS‐NMS, demonstrating the eigenvalue distribution across factors. The plots illustrate the distinct factor separations, with clear declines at the elbow points, confirming the number of meaningful factors retained for each domain. These visualizations support the robustness of the factor structure and align with theoretical expectations for domain‐specific dimensionality.

**Data S1.** Detailed Methodology of Analyses. Comprehensive description of the statistical procedures and analyses used for confirmatory and exploratory factor analyses of the French MDS‐NMS, including estimation methods, rotation strategy, fit indices, and rationale for sample size.

**Table S1.** Demographic data of the French Population. SD, standard deviation; PD, Parkinson's Disease; HY, Hoehn and Yahr.

**Table S2.** Factor loadings and percent variance explained by each factor in the French version of the NMS‐MDS Scale. Factor 1: The primary factor identified for each symptom domain; Symptoms: Specific symptoms assessed within each domain; Loading: The factor loading, indicating the correlation between each symptom and the underlying factor; Percent variance: The percentage of total variance in the symptoms explained by Factor 1 within each domain.

## Data Availability

The data that support the findings of this study are available on request from the corresponding author. The data are not publicly available due to privacy or ethical restrictions.
