# Supplementary material for: Validation of the French Translation of the Movement Disorder Society Non‐Motor Symptoms Scale (MDS‐NMS) in Parkinson's Disease
Source: Mov Disord Clin Pract. 2025 Sep 1;13(2):575–9. doi: 10.1002/mdc3.70323 (PMC12911461; doi:10.1002/mdc3.70323)
Supplement: Supplementary file 1 — Figure S1. Scree plots for each domain of the MDS‐NMS, demonstrating the eigenvalue distribution across factors. The plots illustrate the distinct factor separations, with clear declines at the elbow points, confirming the number of meaningful factors retained for each domain. These visualizations support the robustness of the factor structure and align with theoretical expectations for domain‐specific dimensionality. [file MDC3-13-575-s004.pdf]

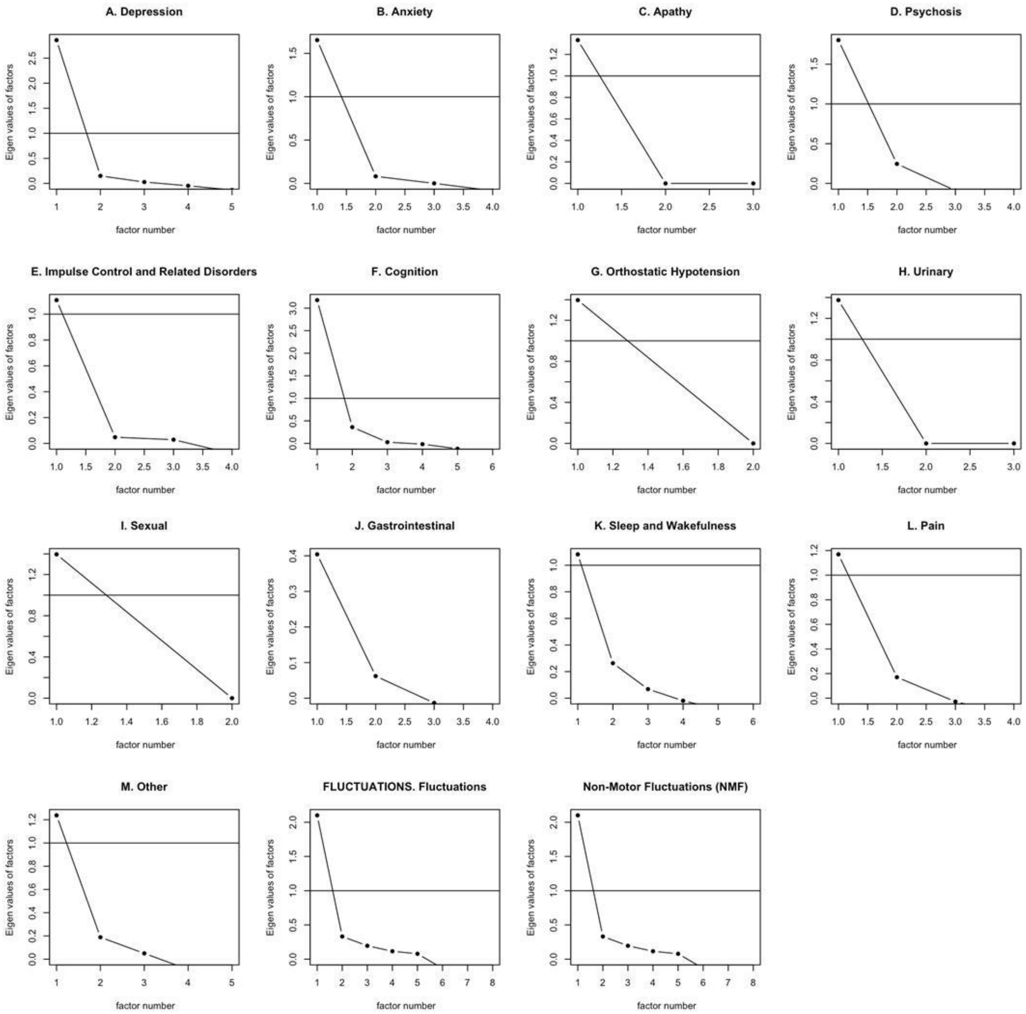
A. Depression
B. Anxiety
C. Apathy
D. Psychosis
E. Impulse Control and Related Disorders
F. Cognition
G. Orthostatic Hypotension
H. Urinary
I. Sexual
J. Gastrointestinal
K. Sleep and Wakefulness
L. Pain
M. Other
FLUCTUATIONS. Fluctuations
Non-Motor Fluctuations (NMF)
Eigen values of factors
factor number
