# Supplementary material for: Validation of the French Translation of the Movement Disorder Society Non‐Motor Symptoms Scale (MDS‐NMS) in Parkinson's Disease
Source: Mov Disord Clin Pract. 2025 Sep 1;13(2):575–9. doi: 10.1002/mdc3.70323 (PMC12911461; doi:10.1002/mdc3.70323)
Supplement: Supplementary file 2 — Data S1. Detailed Methodology of Analyses. Comprehensive description of the statistical procedures and analyses used for confirmatory and exploratory factor analyses of the French MDS‐NMS, including estimation methods, rotation strategy, fit indices, and rationale for sample size. [file MDC3-13-575-s001.docx]

**Methods:**

*Ethical considerations*

The study was conducted within the cohort of the French clinical research network on Parkinson’s disease, NS-PARK cohort(1). All participants gave their informed consent to cohort participation, in line with French ethical guidelines. All patients could express their opposition rights regarding the study through the cohort website, in accordance to EU General Protection Data Regulation rules (<https://parkinson.network/la-cohorte-ns-park>).

*Translation Protocol*

The French translation followed established guidelines by the International Parkinson and Movement Disorder Society (MDS)-Clinical Outcome Assessment (COA) Program. Two bilingual movement disorder specialists independently performed forward translations. Discrepancies were resolved through reconciliation. A separate team conducted the back-translation, which an expert panel reviewed against the original English version.

*Cognitive Pretesting*

Cognitive pretesting is a qualitative approach to assess the task difficulty of an instrument completion for the examiner's and the respondent’s interest, attention span, discomfort, and comprehension. The provisionally approved translation was administered to 10 PD patients for cognitive pre-testing. This phase identifies potentially culturally sensitive or complex items. Based on the initial cognitive pretesting results, another round(s) of translation, back translation and cognitive pretesting could be required. Once cognitive pretesting was completed and no problems were observed, the final translation was approved as “Official Working Document”.

*Study Participants*

The study enrolled 303 PD patients from 10 PD expert centers in France. Inclusion criteria were: a confirmed PD diagnosis based on MDS criteria(2), high level of proficiency in French language, and informed consent. Demographic and clinical data were collected, including age, disease duration, education, and Hoehn and Yahr (HY) stage.

*Factor Analysis*

The R (Version 4.2.0) packages lavaan and psych were used for the primary confirmatory and secondary exploratory factor analyses, respectively, as the variables are categorical. We used an adjusted weighted least square (WLSMV) approach to factor estimation that minimizes the weighted sum of squared differences between observed and estimated correlation matrices not counting diagonal elements. To assist in interpreting the factors, we used an orthogonal CF-VARIMAX rotation that constrains the factors from being uncorrelated.

The sample size for the translation study was based on the need for five subjects per item of the questionnaire to perform the statistical analysis(1). Because there are 60 items (52 MDS-NMS items and eight nonmotor fluctuations items) in the MDS-NMS, a sample of at least 300 patients was required. Any participants with missing values were deleted from the analysis of that domain only. Thus, the sample size from domain to domain could vary. Data without patient names or medical record numbers were transferred to the analytic team via a secure website.

Statistical indices were defined as follows: eigenvalues reflected the variance explained (values >1 considered meaningful); CMIN (Chi-Square) measured the discrepancy between observed and expected covariances (lower values indicated better fit); DF represented free parameters; RMSEA assessed fit per degree of freedom (<0.08 acceptable); and CFI compared the model to an independent one (>0.90 indicating good fit).

Primary Analysis

For the primary analysis of the French data, we conducted a confirmatory factor analysis (CFA) to determine if the factor structure for the English language MDS-NMS (2) could be confirmed in data collected using the French translation. This was the primary question of interest. The CFA was conducted separately for each of the 14 subscales (13 domains of the MDS-NMS plus the NMF subscale) with the French data constrained to fall into the factors defined in the English language data. We evaluated the CFA results based on the CFI. According to the protocol, to establish a successful translation and to designate that translation as an official MDS translation of the MDS-NMS, we required that the CFI for each Part of the translated MDS-NMS be 0.90 or greater relative to the English language version. Mean and variance-adjusted WLSMV estimators were used to confirm model fit.

Secondary Analysis

As a secondary analysis we conducted an exploratory factor analysis (EFA) for each of the 14 subscales of the French version of the MDS-NMS to explore the underlying factor structure without the constraint of a pre-specified factor structure. Once the factors are chosen, an item was retained in a factor if the factor loading for that item was 0.40 or greater. To assist ininterpreting the factors, an orthogonal CF-VARIMAX rotation was used, which sets the factors as uncorrelated.

**References:**

1. Mariani LL, Doulazmi M, Chaigneau V, Brefel-Courbon C, Carrière N, Danaila T, et al. Descriptive analysis of the French NS-Park registry: Towards a nation-wide Parkinson’s disease cohort? Parkinsonism Relat Disord. 2019 Jul;64:226–34.

2. Postuma RB, Berg D, Stern M, Poewe W, Olanow CW, Oertel W, et al. MDS clinical diagnostic criteria for Parkinson’s disease: MDS-PD Clinical Diagnostic Criteria. Mov Disord. 2015 Oct;30(12):1591–601.
