# Supplementary material for: Validation of the French Translation of the Movement Disorder Society Non‐Motor Symptoms Scale (MDS‐NMS) in Parkinson's Disease
Source: Mov Disord Clin Pract. 2025 Sep 1;13(2):575–9. doi: 10.1002/mdc3.70323 (PMC12911461; doi:10.1002/mdc3.70323)
Supplement: Supplementary file 3 — Table S1. Demographic data of the French Population. SD, standard deviation; PD, Parkinson's Disease; HY, Hoehn and Yahr. [file MDC3-13-575-s002.docx]

| **Supplementary Table 1. Demographic data of French population** | |
| --- | --- |
| Variable |  |
| n | 303 |
| Male, N (%) | 194 (64.0) |
| Age, years | 62.3 (10.94) |
| Mean (SD) |  |
| PD diagnosis, years | 8.17 (7.81) |
| Mean (SD) |  |
| Education, years | 13.03 (4.44) |
| Mean (SD) |  |
| HY stage, N (%) |  |
| 0 | 5 (1.65) |
| 1 | 63 (20.79) |
| 1.5 | 5 (1.65) |
| 2 | 175 (57.76) |
| 2.5 | 11 (3.63) |
| 3 | 29 (9.57) |
| 4 | 5 (1.65) |
| Missing data | 10 (3.30) |
|  | |
