# Supplementary material for: Validation of the French Translation of the Movement Disorder Society Non‐Motor Symptoms Scale (MDS‐NMS) in Parkinson's Disease
Source: Mov Disord Clin Pract. 2025 Sep 1;13(2):575–9. doi: 10.1002/mdc3.70323 (PMC12911461; doi:10.1002/mdc3.70323)
Supplement: Supplementary file 4 — Table S2. Factor loadings and percent variance explained by each factor in the French version of the NMS‐MDS Scale. Factor 1: The primary factor identified for each symptom domain; Symptoms: Specific symptoms assessed within each domain; Loading: The factor loading, indicating the correlation between each symptom and the underlying factor; Percent variance: The percentage of total variance in the symptoms explained by Factor 1 within each domain. [file MDC3-13-575-s003.docx]

**Supplementary Table 2.** **Factor loadings and percent variance explained by each factor in the French version of the NMS-MDS Scale.**

| **Factor** | **Symptoms** | **Loading** |
| --- | --- | --- |
| **Factor** | **Symptoms** | **Loading** |
| **A: Depression** | | |
| **Factor 1** | *Percent variance* | *57.2* |
|  | 1. Felt sad or depressed? | 0.82 |
|  | 2. Had difficulty experiencing pleasure? | 0.72 |
|  | 3. Felt hopeless? | 0.72 |
|  | 4. Had negative thoughts about yourself? | 0.74 |
|  | 5. Felt that life is not worth living? | 0.77 |
| **B: Anxiety** | | |
| **Factor 1** | *Percent variance* | *41.4* |
|  | 1. Felt worried? | 1.00 |
|  | 2. Felt nervous? | 0.59 |
|  | 3. Had panic or anxiety attacks? | 0.44 |
| **Not loaded** | 4. Been worried about being in public or in social situations? |  |
| **C: Apathy** | | |
| **Factor 1** | *Percent variance* | *44.1* |
|  | 1. Had a reduced motivation to start day-to-day activities? | 0.61 |
|  | 2. Had a reduced interest in talking to people? | 0.88 |
|  | 3. Had a reduction in experiencing emotions? | 0.42 |
| **D: Psychosis** | | |
| **Factor 1** | *Percent variance* | *45.0* |
|  | 1. Sensed things or people in the margins of your visual field? (passage or presence phenomena) | 0.65 |
|  | 2. Visually misinterpreted an actual object? (illusions) | 0.73 |
|  | 3. Seen, heard, felt, tasted, or smelled things that other people did not? (hallucinations) | 0.69 |
|  | 4. Believed things to be true that others did not? (e.g., delusions of persecution, jealousy, or misidentification) | 0.61 |
| **E: Impulse Control and Related Disorders** | | |
| **Factor 1** | *Percent variance* | *23.2* |
|  | 1. Had an increase in gambling, sexual, buying, or eating behaviors? | 0.89 |
| **Not loaded** | 2. Had an increase in other behaviors (e.g., internet use, hobbies, artistic activities, writing, hoarding)? |  |
|  | 3. Repeatedly handled objects without any purpose? (punding) |  |
|  | 4. Routinely taken more anti-parkinsonian medications than prescribed? (dopamine dysregulation syndrome) |  |
| **F: Cognition** | | |
| **Factor 1** | *Percent variance* | *53.0* |
|  | 1. Had difficulty remembering things? | 0.82 |
|  | 2. Had difficulty learning new things? | 0.76 |
|  | 3. Had difficulty keeping focus or paying attention? | 0.80 |
|  | 4. Had difficulty finding words or expressing ideas? | 0.72 |
|  | 5. Had difficulty planning or carrying out complex tasks, not due to motor problems? (executive abilities) | 0.75 |
|  | 6. Had difficulty judging the position of things? (visuospatial abilities) | 0.45 |
| **G: Orthostatic Hypotension** | | |
| **Factor 1** | *Percent variance* | *69.8* |
|  | 1. Felt lightheaded or fainted when changing position? | 0.84 |
|  | 2. Had dizziness or weakness upon standing? | 0.84 |
| **H: Urinary** | | |
| **Factor 1** | *Percent variance* | *45.6* |
|  | 1. Had an urgent need to empty bladder? (urinary urgency) | 0.83 |
|  | 2. Had to empty bladder more than every 2 hours? (urinary frequency) | 0.65 |
|  | 3. Had to empty bladder more than twice overnight? (nocturia) | 0.51 |
| **I: Sexual** | | |
| **Factor 1** | *Percent variance* | *69.7* |
|  | 1. Had decreased sexual drive or interest in sex? | 0.84 |
|  | 2. Had difficulty with sexual arousal (e.g., erectile dysfunction or vaginal dryness) or sexual performance not related to motor problems (e.g., not related to Parkinson’s rigidity)? | 0.84 |
| **J: Gastrointestinal** | | |
| **Factor 1** | *Percent variance* | *10.1* |
| **Not loaded** | 1. Had any drooling of saliva? |  |
|  | 2. Had difficulty swallowing? |  |
|  | 3. Had nausea or felt sick in the stomach? |  |
|  | 4. Had constipation? (defined as < 3 bowel movements/week) |  |
| **K: Sleep and Wakefulness** | | |
| **Factor 1** | *Percent variance* | *18.0* |
|  | 3. Dozed off or fallen asleep unintentionally during waking hours? (e.g., during conversation, at mealtimes, or while driving, watching television; excessive daytime sleepiness) | 0.42 |
|  | 4. Had an irresistible urge to move legs or arms when sitting or lying down, which improved with movement? (restlessness) | 0.48 |
|  | 5. Had any involuntary jerky movements in arms or legs during sleep or while resting? (periodic limb movements) | 0.51 |
|  | 6. Woken at night due to snoring, gasping, or difficulty with breathing? | 0.41 |
| **Not loaded** | 1. Had difficulty falling asleep or staying asleep? (insomnia) |  |
|  | 2. Acted out dreams while asleep, such as shouting, flailing arms, punching, or running movements? (REM sleep behavior) |  |
| **L: Pain** | | |
| **Factor 1** | *Percent variance* | *29.2* |
|  | 1. Had muscle, joint, or back pain? | 0.64 |
|  | 2. Had a deep or dull aching pain within the body? | 0.72 |
| **Not loaded** | 3. Had pain due to abnormal twisting movements of arms or legs or body, often present in the early morning period? (dystonia) |  |
|  | 4. Had other types of pain? (e.g., nocturnal pain, orofacial pain) |  |
| **M: Other** | | |
| **Factor 1** | *Percent variance* | *24.5* |
|  | 3. Felt excessively physically tired? (physical fatigue) | 0.84 |
|  | 4. Felt excessively mentally tired? (mental fatigue) | 0.65 |
| **Not loaded** | 1. Had an unintentional weight loss? |  |
|  | 2. Had a decrease in sense of smell? (impaired olfaction) |  |
|  | 5. Had excessive sweating not related to temperature? |  |
|  | **Non-Motor Fluctuations (NMF)** |  |
| **Factor 1** | *Percent variance* | *26.3* |
|  | 1. Depression (as listed in Subscale A) | 0.67 |
|  | 2. Anxiety (as listed in Subscale B) | 0.66 |
|  | 3. Thinking or cognitive abilities (as listed in Subscale F) | 0.53 |
|  | 6. Pain (as listed in Subscale L) | 0.51 |
|  | 7. Fatigue (as listed in Subscale M, items 3 and 4) | 0.61 |
| **Not loaded** | 4. Bladder symptoms (as listed Subscale H) |  |
|  | 5. Restlessness (as listed in Subscale K, item 4) |  |
|  | 8. Excessive sweating (as listed in Subscale M, item 5) |  |
| *The number of factors is selected based on scree plots in Figure 1. | | |
